# Acetylharpagide Protects Mice from *Staphylococcus Aureus*-Induced Acute Lung Injury by Inhibiting NF-κB Signaling Pathway

**DOI:** 10.3390/molecules25235523

**Published:** 2020-11-25

**Authors:** Zhaoxin Zhang, Yun Wang, Yating Shan, Wu Yin

**Affiliations:** The State Key Lab of Pharmaceutical Biotechnology, College of life Sciences, Nanjing University, Nanjing 210023, China; zhaoxinzhang1989@126.com (Z.Z.); wy1803556196@163.com (Y.W.); shanyating340303@126.com (Y.S.)

**Keywords:** acute lung injury, *Staphylococcus aureus*, acetylharpagide, NF-κB signaling

## Abstract

*Staphylococcus aureus* (*S. aureus*)-induced acute lung injury (ALI) is a serious disease that has a high risk of death among infants and teenagers. Acetylharpagide, a natural compound of *Ajuga decumbens* Thunb. (family Labiatae), has been found to have anti-tumor, anti-inflammatory and anti-viral effects. This study investigates the therapeutic effects of acetylharpagide on *S. aureus*-induced ALI in mice. Here, we found that acetylharpagide alleviated *S. aureus*-induced lung pathological morphology damage, protected the pulmonary blood-gas barrier and improved the survival of *S. aureus*-infected mice. Furthermore, *S. aureus*-induced myeloperoxidase (MPO) activity of lung homogenate and pro-inflammatory factors in bronchoalveolar lavage (BAL) fluid were suppressed by acetylharpagide. Mechanically, acetylharpagide inhibited the interaction between polyubiquitinated receptor interacting protein 1 (RIP1) and NF-κB essential modulator (NEMO), thereby suppressing NF-κB activity. In summary, these results show that acetylharpagide protects mice from *S. aureus*-induced ALI by suppressing the NF-κB signaling pathway. Acetylharpagide is expected to become a potential treatment for *S. aureus*-induced ALI.

## 1. Introduction

Acute lung injury (ALI) is caused by severe infection, trauma and sepsis, and is mainly shown as non-cardiogenic pulmonary edema, progressive dyspnea and refractory hypoxemia [1,2]. In some circumstances, ALI deteriorates into acute respiratory distress syndrome (ARDS) [3]. After years of basic and clinical research, the diagnosis and treatment of ALI are improving day by day. However, due to its complicated pathogenesis and poor treatment effect, the mortality rate still remains high. It is generally considered that microbial infection is the main cause of ALI [4]. *Staphylococcus aureus* (*S. aureus*) is regarded as a highly infectious Gram-positive bacterium for ALI [5,6]. *S. aureus* infection activates the host’s immune system, causing infiltration of inflammatory cells (like neutrophils and macrophages), inducing expression of cytokines and chemokines, leading to uncontrollable inflammatory response [7]. Many studies have found that excessive lung inflammation was the main cause of ALI and alleviating inflammation was an effective way to treat ALI [8,9,10]. Recently, many studies have reported that traditional Chinese medicines, such as *Scutellaria baicalensis, Lonicera japonica, Ajuga decumbens* and *Forsythia suspensa* can effectively relieve inflammation and defense against infection [11,12,13,14].

*Ajuga decumbens* Thunb. (family Labiatae) is a common traditional Chinese medicine for dispelling internal heat and toxic material. It can be used for respiratory tract infections, such as tonsillitis, pharyngitis and bronchitis [15]. Its active ingredients have many biological effects such as anti-inflammatory, bacteriostatic, immune function regulation, anti-fibrosis, anti-tumor and so on [16,17]. Acetylharpagide is one of the wonderful active ingredients derived from *Ajuga decumbens* and was first discovered to have vasoconstrictive activity in guinea pig [18]. Recently, some studies have reported that acetylharpagide had outstanding antibacterial, anti-inflammatory, and antiviral activities [19,20,21]. However, the effects of acetylharpagide on *S. aureus*-induced pneumonia are still unclear.

**Figure 1:**
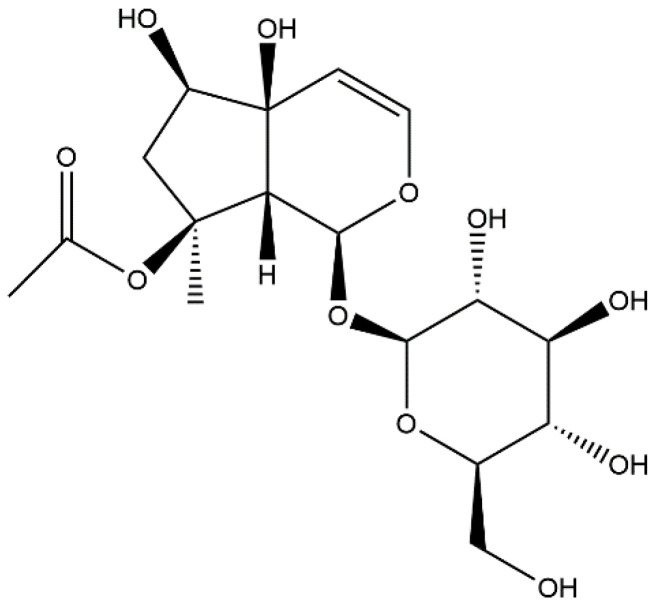
The chemical structure of acetylharpagide.

Acetylharpagide is one of the active ingredients derived from *Ajuga decumbens* [13,18,19,20,21], a common traditional Chinese medicine for dispelling internal heat and toxic material [15]. Its chemical formula is C_17_H_26_O_11_ and its structure was drawn with chemdraw software (Figure 1). Acetylharpagide is a member of ridoid glycosides. Iridoid glycosides are usually divided into nine-carbon skeleton iridoid glycoside type, ten-carbon skeleton iridoid glycoside type and secoiridoid glycoside type. Acetylharpagide belongs to the nine-carbon skeleton iridoid glycoside type (Figure 1). Iridoid glycosides are one of the most common ingredients in traditional Chinese medicines. For example, the well-known iridoid glycosides geniposide, catalpol, picroside II and gentiopicroside/gentiopicrin are the indicator components of traditional Chinese medicines *Gardenia jasminoides*, *Rehmannia glutinosa*, *Picrorhiza scrophulariiflora* and *Gentiana scabra*, respectively [22,23,24,25]. Iridoid glycosides have a wide range of pharmacological effects, the most prominent of which is anti-inflammatory activity [26,27,28]. Studies have found that the anti-inflammatory effects of iridoid glycosides are mostly related to the NF-κB pathway and the mitogen-activated protein kinase (MAPK) pathway. Yu et al. found geniposide that was isolated from *Gardenia jasminoides* attenuated *S. aureus*-induced pneumonia by supressing NF-κB activation [29]. He et al. found that scandoside isolated from *Hedyotis diffusa* reduced inflammation by blocking NF-κB and MAPK signaling pathways in lipopolysaccharide (LPS)-stimulated macrophages [30]. Liang et al. found that gardenoside had a protective effect on free fatty acid-induced steatosis in HepG2 hepatocytes, and the mechanism may be to reduce the secretion of pro-inflammatory factors by inhibiting intracellular NF-κB activity [31].

In this study, we found that acetylharpagide relieved *S. aureus*-induced pneumonia and lung injury, and improved the survival of *S. aureus*-infected mice. Mechanically, acetylharpagide inhibited the interaction between polyubiquitinated receptor interacting protein 1 (RIP1) and NF-κB essential modulator (NEMO), thereby inhibiting NF-κB activity and cytokine production. In summary, acetylharpagide protects mice from *S. aureus*-induced pneumonia and lung injury by suppressing NF-κB signaling pathways and acetylharpagide is expected to become a potential treatment for *S. aureus*-induced ALI.

## 2. Results

### 2.1. Acetylharpagide Protects Mice from S. aureus-Induced ALI

**Figure 2:**
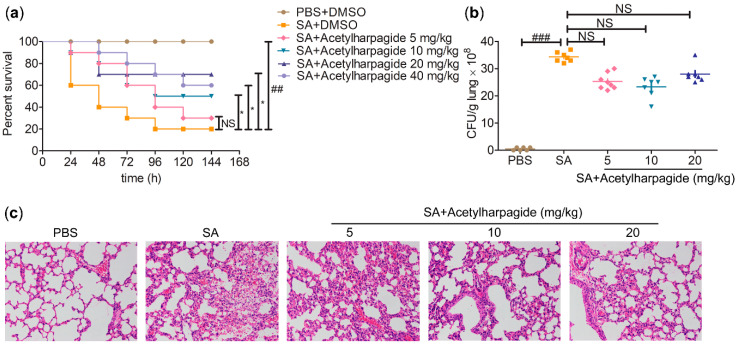
Acetylharpagide protected mice from *Staphylococcus aureus* (SA)-induced acute lung injury (ALI). (**a**) Acetylharpagide was injected intraperitoneally daily from day 0 to 5. The survival of *S. aureus*-infected mice with or without acetylharpagide was recorded. Kaplan-meier curves and log rank test were used to compare mortality rates, ^##^ *p* < 0.01 vs. phosphate buffered saline (PBS)+DMSO group, * *p* < 0.05 vs. SA+DMSO group, *n* = 10; (**b**) The bacterial loads of the lung homogenate were measured at 12 h after *S. aureus* infection. The data were presented as mean ± SD, ^###^ *p* < 0.001 vs. PBS+DMSO group, *n* = 6–8; (**c**) Hematoxylin and eosin staining of mouse lung tissues that infected with *S. aureus* for 12 h, ×200.

Many studies have reported that acetylharpagide has outstanding antibacterial, anti-inflammatory and antiviral activities [19,20,21]. To explore whether acetylharpagide takes part in *S. aureus*-induced ALI and how it works, we established the ALI mice model infected with *S. aureus*. Mice were inoculated *S. aureus* directly into the trachea and intraperitoneally injected with 5 mg/kg, 10 mg/kg, 20 mg/kg or 40 mg/kg of acetylharpagide 2 h prior to *S. aureus* infection. We found that acetharpagide improved the survival rate of *S. aureus* infected mice (Figure 2a). Within a certain range, the improved effect gradually enhanced with the increase of dose, of which 20 mg/kg had the best effect. *S. aureus* infection significantly increased the bacterial loads in the lungs, but acetharpagide seemed to have no effect on bacterial clearance (Figure 2b). Hematoxylin and eosin (H&E) staining showed that after *S. aureus* infection, the alveolar walls were thickened, broken or even collapsed, and many inflammatory cells infiltrated in the alveolar cavity. There was obvious edema and hyperemia around the bronchi, and accumulation of edema fluid could be seen in the alveolar cavity. Acetylharpagide pretreatment reduced *S. aureus*-induced lung damage, and the effect gradually enhanced with the increase of dose. The lung tissue structure was clear, the alveolar wall was not thickened and the infiltration of inflammatory cells and the accumulation of edema fluid in the alveolar cavity were reduced (Figure 2c). Thus, these results suggest that acetylharpagide protects mice from *S. aureus*-induced ALI.

### 2.2. Acetylharpagide Protects the Pulmonary Blood-Gas Barrier in S. aureus-Induced ALI

**Figure 3:**
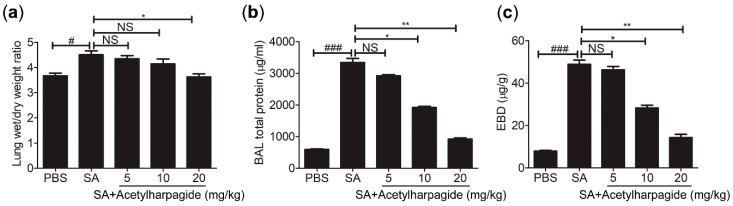
Acetylharpagide relieved *S. aureus* (SA)-induce pulmonary vascular permeability and edema. (**a**) The lung wet/dry ratio was detected at 12 h after *S. aureus* infection; (**b**) Bronchoalveolar lavage (BAL) fluid was collected at 12 h after *S. aureus* infection and bicinchoninic acid (BCA) protein assay was performed to measure the total protein concentration; (**c**) The concentration of evans blue dye (EBD) in the lung tissue was examined at 12 h after *S. aureus* infection. The data were presented as mean ± SD, ^#^ *p* < 0.05, ^###^ *p* < 0.001 vs. PBS+DMSO group, * *p* < 0.05, ** *p* < 0.01 vs. SA+DMSO group, *n* = 6–8.

The pulmonary gas-blood barrier is a membrane that is used to exchange oxygen in the alveoli and carbon dioxide in the capillaries of the lungs. Its integrity is critical to gas exchange and to prevent the backflow of substances from the blood into the interstitial and alveolar cavities. The destruction of its integrity would result in increased permeability and pulmonary edema, which are the core pathological changes of ALI [32]. In our study, lung wet/dry ratio was examined to evaluate lung water content, and the concentration of evans blue dye in the lung tissue and the total protein in bronchoalveolar lavage (BAL) fluid were determined to evaluate vascular leak (Figure 3). We found that mice infected with *S. aureus* showed increased lung wet/dry ratio (Figure 3a), elevated total protein concentration in BAL fluid (Figure 3b) and aggravated evans blue dye extravasation (Figure 3c). However, acetylharpagide pretreatment significantly attenuated pulmonary edema and vascular leakage, and the effect gradually enhanced with the increase of dose (Figure 3). Thus, these results suggest that acetylharpagide protects the pulmonary blood-gas barrier in *S. aureus*-induced ALI.

### 2.3. Acetylharpagide Attenuates S. aureus-Induced Inflammation

**Figure 4:**
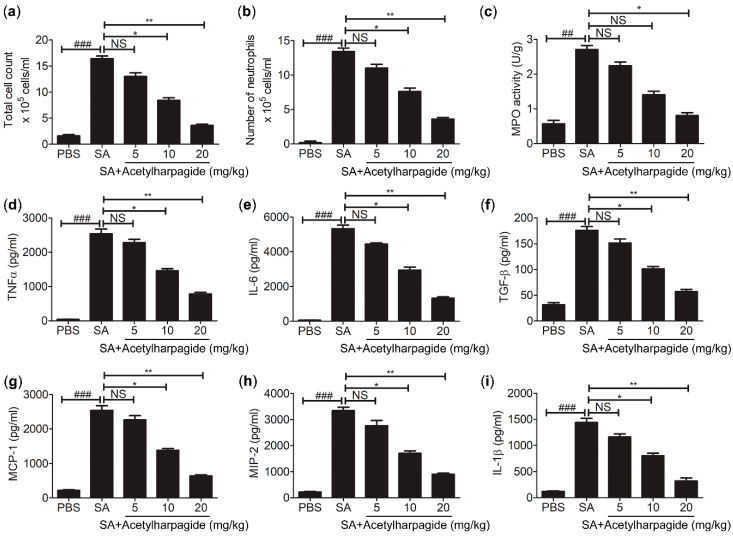
Acetylharpagide attenuated *S. aureus* (SA)-induced inflammation. (**a**,**b**) The number of total cells and neutrophils in BAL fluid were counted at 12 h after *S. aureus* infection; (**c**) The myeloperoxidase (MPO) activity of lung homogenate was measured by ELISA at 12 h after *S. aureus* infection; (**d**–**i**) ELISA was performed to measure the concentrations of TNF-α (**d**), IL-6 (**e**), TGF-β (**f**), MCP-1 (**g**), MIP-2 (**h**) and IL-1β (**i**) in BAL fluid at 12 h after *S. aureus* infection. The data were presented as mean ± SD, ^##^ *p* < 0.01, ^###^ *p* < 0.001 vs. PBS+DMSO group, * *p* < 0.05, ** *p* < 0.01 vs. SA+DMSO group, *n* = 6–8.

In the process of *S. aureus* infection, neutrophil is one of the most important members of the innate immune system, and it is also one of the first effector cells recruited to the inflammatory area to exert immune defense [33]. Neutrophil contains a large amount of cytotoxic substances. Although these cytotoxic substances can kill pathogenic microorganisms, they may also aggravate inflammatory reactions and lung injury [34]. We found that in the BAL fluid of *S. aureus*-infected mice, total cells and neutrophils had greatly increased, and the majority of the total cells were neutrophils. However, acetylharpagide pretreatment reduced the number of total cells and neutrophils in BAL fluid that induced by *S. aureus* infection (Figure 4a,b), and the effect gradually improved with the increase of dose. Consistently, myeloperoxidase (MPO) activity, a marker indicating the degree of neutrophil infiltration, was elevated in *S. aureus*-infected mice, but decreased by acetylharpagide pretreatment (Figure 4c). Enzyme-linked immunosorbent assays (ELISA) results revealed that *S. aureus*-induced cytokines TNF-α, IL-6, TGF-β, MCP-1, MIP-2 and IL-1β in BAL fluid were also reduced by acetylharpagide pretreatment, and the effect gradually improved with the increase of dose (Figure 4d–i). Thus, these results suggest that acetylharpagide attenuates *S. aureus*-induced inflammation.

### 2.4. Acetylharpagide Inhibits S. aureus-Induced Pro-Inflammatory Cytokines in Macrophages

**Figure 5:**
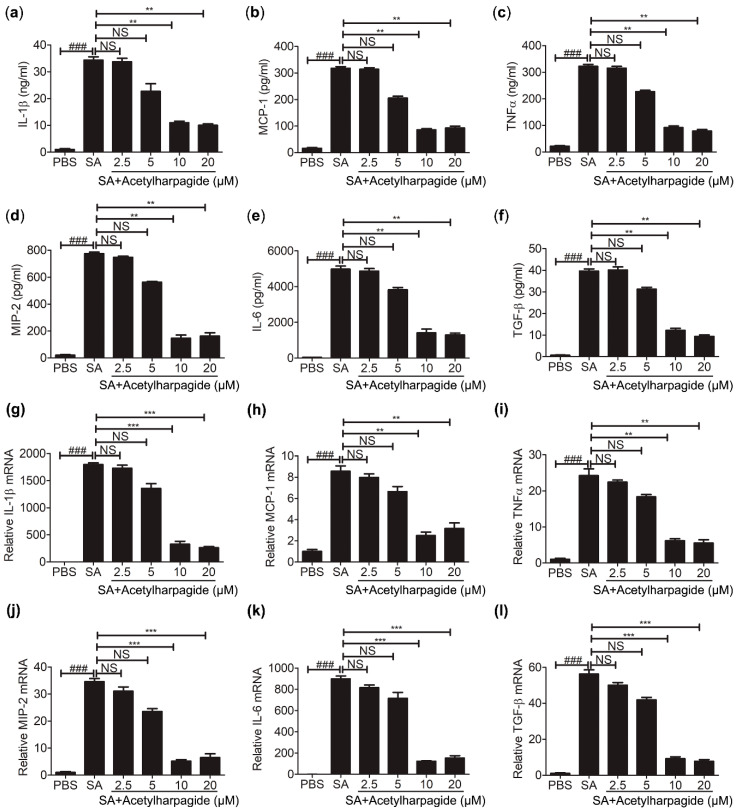
Acetylharpagide inhibited *S. aureus* (SA)-induced pro-inflammatory cytokines in macrophages. Raw264.7 cells were pretreated with acetylharpagide or DMSO for 2 h, then stimulated with *S. aureus* (multiplicity of infection (MOI), 10:1) for 2 h. (**a**–**f**) The culture medium was harvested and determined for the concentrations of IL-1β (**a**), MCP-1 (**b**), TNFα (**c**), MIP-2 (**d**), IL-6 (**e**) and TGF-β (**f**) by ELISA. (**g**–**l**) The mRNA expression of IL-1β (**g**), MCP-1 (**h**), TNFα (**i**), MIP-2 (**j**), IL-6 (**k**) and TGF-β (**l**) was determined by qPCR. The data were presented as mean ± SD, ^###^ *p* < 0.001 vs. PBS+DMSO group, ** *p* < 0.01, *** *p* < 0.001 vs. SA+DMSO group, *n* = 3.

Macrophages are an essential member of the innate immune system and play important roles in cytokine secretion and pathogen elimination [35]. To study the anti-inflammatory mechanisms of acetylharpagide against *S. aureus* infection, we cultured Raw264.7 cells (mouse leukemia cells of monocyte macrophage) in vitro. Cells were pretreated with acetylharpagide or DMSO followed by *S. aureus* infection. The culture medium was harvested for determining the concentration of cytokines. ELISA results revealed that *S. aureus*-induced cytokines IL-1β, MCP-1, TNF-α, MIP-2, IL-6 and TGF-β were decreased by acetylharpagide pretreatment. The inhibitory effect gradually enhanced with the increase of dose, and reached a maximum at 10 μM (Figure 5a–f). Consistently, acetylharpagide pretreatment inhibited the mRNA expression of these pro-inflammatory factors in Raw264.7 cells (Figure 5g–l). Thus, these results predict that acetylharpagide inhibits *S. aureus*-induced pro-inflammatory cytokines in macrophages.

### 2.5. Acetylharpagide Suppresses S. aureus-Induced NF-κB Activity

**Figure 6:**
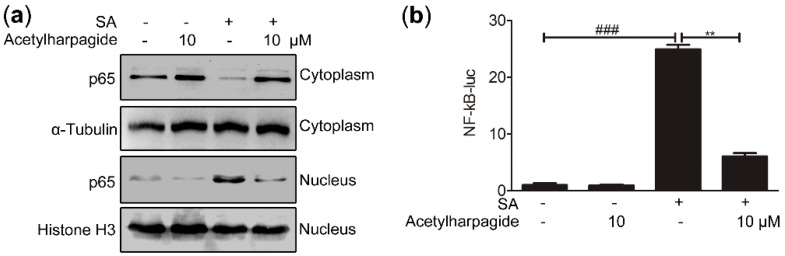
Acetylharpagide suppressed *S. aureus* (SA)-induced NF-κB activity. (**a**) Raw264.7 cells were pretreated with acetylharpagide or DMSO for 2 h, then stimulated with *S. aureus* (multiplicity of infection (MOI), 10:1) for 2 h. Nuclear and cytoplasmic proteins were extracted and detected for the expression of p65 by western blot; (**b**) NF-κB luciferase reporter plasmid was transfected into Raw264.7 cells. After 24 h, cells were stimulated as in A. Luciferase assays were carried out with the dual-luciferase reporter assay system, and the ratio of firefly luminescence to renilla luminescence was evaluated to reflect NF-κB activity. The relative luciferase activity normalized to the control group is shown. The data were presented as mean ± SD, ^###^ *p* < 0.001 vs. PBS+DMSO group, ** *p* < 0.01 vs. SA+DMSO group, *n* = 3.

The above results indicate that acetylharpagide has anti-inflammatory effects on *S. aureus* infection. However, the mechanism is still poorly understood. NF-κB signaling pathways play important roles in inflammatory and immune response [36,37,38]. Some studies have reported that inhibition of this pathway relieved *S. aureus*-induced pneumonia and lung injury [39,40,41,42]. Since the secretion of cytokines requires nuclear translocation of the transcription factor NF-κB, we examined the effects of acetylharpagide on NF-κB nuclear translocation. The cytoplasmic and nuclear proteins were collected and western blot was carried out to investigate the expression of p65 in the cytoplasm and nucleus. We found that *S. aureus* infection induced the transfer of p65 from the cytoplasm into the nucleus, whereas acetylharpagide pretreatment reduced this translocation (Figure 6a). To measure the effect of acetylharpagide on the activity of transcription factor NF-κB, NF-κB luciferase reporter plasmid was transfected into Raw264.7 cells, and the following *S. aureus*-induced luciferase activity in the absence or presence of acetylharpagide was detected with the dual-luciferase reporter assay system. The results revealed that acetylharpagide pretreatment significantly decreased the *S. aureus*-induced NF-κB activity (Figure 6b). Thus, these results suggest that acetylharpagide suppresses *S. aureus*-induced NF-κB activity in Raw264.7 cells.

### 2.6. Acetylharpagide Inhibits the Interaction between Polyubiquitinated RIP1 and NEMO upon S. aureus Infection

**Figure 7:**
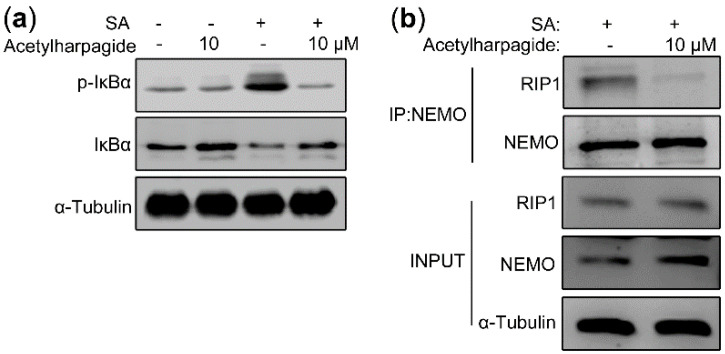
Acetylharpagide inhibited the interaction between polyubiquitinated RIP1 and NEMO upon *S. aureus* (SA) stimulation. (**a**) Raw264.7 cells were pretreated with acetylharpagide or DMSO for 2 h, then stimulated with *S. aureus* (multiplicity of infection (MOI), 10:1) for 2 h. The expressions of IκBα and phosphorylated IκBα in cellular lysates were analyzed by western blot; (**b**) Raw264.7 cells were stimulated as in A. Cellular lysates were subjected to immunoprecipitation with an anti-NEMO antibody followed by immunoblotting with anti-NEMO and anti-RIP1 antibodies.

In the resting state, NF-κB was trapped in the cytoplasm by the inhibitory protein IκBα. Once activated, IκBα was phosphorylated by IKKβ and degraded by 26S proteasome, thus releasing NF-κB to the nucleus, initiating the transcription of inflammatory cytokines and inducing inflammation [43,44]. Therefore, we assessed the effect of acetylharpagide on *S. aureus*-induced IκBα phosphorylation and degradation. Western blot analysis showed that *S. aureus* elevated the level of IκBα phosphorylation, and the phosphorylated IκBα was then degraded by the 26S proteasome, resulting in a decrease in the level of total IκBα. However, acetylharpagide pretreatment inhibited the phosphorylation of IκBα, thereby inhibiting the degradation of IκBα, resulting in an increase in the level of total IκBα. These results indicate that acetylharpagide pretreatment markedly inhibits *S. aureus*-induced IκBα phosphorylation and degradation in Raw264.7 cells (Figure 7a). We next studied the target or molecular mechanism by which acetylharpagide regulates NF-κB signaling. Co-immunoprecipitation results revealed that *S. aureus*-induced interaction between NEMO and polyubiquitinated RIP1 was inhibited by acetylharpagide pretreatment in Raw264.7 cells (Figure 7b). These results predict that inhibition of the interaction between NEMO and polyubiquitinated RIP1 by acetylharpagide prevents IκBα phosphorylation and degradation, and ultimately inhibits NF-κB activity.

## 3. Discussion

*S. aureus* infections lead to an acute inflammatory response, which clears invading pathogens, repairs damaged tissues and maintains homeostasis [45,46]. However, excessive or uncontrolled inflammation would cause or aggravate tissue and organ injury [47]. At present, there are mainly glucocorticoids and nonsteroidal anti-inflammatory drugs, but long-term use of glucocorticoids has serious side effects, such as reducing the body’s resistance to pathogenic microorganisms, osteoporosis, epilepsy, etc. [48]. In nonsteroidal anti-inflammatory drugs, non-selective cyclooxygenase (COX) inhibitors would cause serious gastric ulcer complications [49], and selective COX-2 inhibitors would elevate the hazard of cardiovascular disease [50]. Because of these side effects of glucocorticoids and nonsteroidal anti-inflammatory drugs, finding novel treatments against *S. aureus* infections is urgent. Recently, many studies have reported that traditional Chinese medicines can effectively relieve *S. aureus*-induced inflammation and defend against infections [51,52,53]. In our study, we found that acetylharpagide, an iridoid glycoside isolated from *Ajuga decumbens*, attenuated the inflammation in *S. aureus*-induced ALI by suppressing NF-κB signaling pathway, thus reducing lung damage and improving the survival of *S. aureus*-infected mice.

ALI mainly presents as lung structural damage, inflammatory cell infiltration and pulmonary edema [54,55]. The wet/dry weight ratio is a marker of pulmonary edema. We found that the wet weight of the lungs increased after *S. aureus* infection, but greatly lightened with acetylharpagide treatment, indicating that acetylharpagide could relieve pulmonary edema effectually. In addition, MPO activity, a marker indicating neutrophil infiltration, was elevated in *S. aureus*-infected mice, but significantly decreased by acetylharpagide treatment, suggesting that acetylharpagide could prevent the infiltration of neutrophils in lungs. Furthermore, H&E staining showed that acetylharpagide pretreatment reduced *S. aureus*-induced lung structural damage. These results demonstrate that acetylharpagide protects mice from *S. aureus*-induced ALI.

Cytokines are small molecular polypeptides or glycoproteins secreted by immune cells. A lot of pro-inflammatory cytokines play important roles in regulating immune response and inflammation [56]. In our study, we found *S. aureus* infection triggered severe pneumonia, which caused a great rise in pro-inflammatory factors, such as IL-1β, IL-6, TGF-β, MIP-2, MCP-1 and TNF-α in bronchoalveolar lavage fluid. However, acetylharpagide pretreatment inhibited *S. aureus*-induced pro-inflammatory cytokines and attenuated *S. aureus*-induced inflammation.

Macrophages are an essential member of immune cells. They have powerful functions of phagocytosis, migration and secretion of pro-inflammatory cytokines [35]. Therefore, Raw264.7 murine macrophages were used in vitro to investigate the activity and mechanism of acetylharpagide on *S. aureus* infection. In our study, Raw264.7 cells were pretreated with acetylharpagide or DMSO followed by *S. aureus* infection. The culture medium was harvested for determining the concentration of cytokines. ELISA results revealed that *S. aureus*-induced cytokines IL-1β, MCP-1, TNF-α, MIP-2, IL-6 and TGF-β were decreased by acetylharpagide pretreatment. Consistently, acetylharpagide pretreatment inhibited the mRNA expression of these pro-inflammatory factors. These results predict that acetylharpagide inhibits the secretion of pro-inflammatory factors that is induced by *S. aureus* in macrophages.

The canonical NF-κB signaling pathway, which plays important roles in inflammation and immune response, has been considered a valuable target for drug design [36,37,38]. Because of the several levels, NF-κB signaling pathway can be targeted at multiple points including phosphatase, ubiquitination, nuclear translocation, DNA binding, protein acetyltransferase and methyltransferase [57]. Some studies have shown that many drugs inhibit the NF-κB signaling pathway by inhibiting the phosphorylation of IκB or the nuclear transport of p65 [58,59]. In our study, we found *S. aureus*-induced IκBα phosphorylation and p65 translocation from the cytoplasm into the nucleus were reduced by acetylharpagide pretreatment. In addition, *S. aureus*-induced luciferase activity of NF-κB reporter plasmid was also reduced by acetylharpagide pretreatment.

In the canonical NF-κB signaling pathway, once the ligands, such as lipopolysaccharide or cytokines, bind to the related receptor, it will cause a configuration change in the latter, and induce the polyubiquitination of RIP1 at Lys-377. This polyubiquitin chains act as a scaffold to recruit TAK1 and IKK complexes via their adaptor proteins TAB2/3 and NEMO, respectively [60,61]. After that, TAK1 phosphorylates IKKβ and activates the IκB kinase IKK, which phosphorylates the inhibitor of NF-κB (IκB) and makes its 26S proteasome degrade, thus releasing NF-κB to the nucleus to start up the expression of target genes, such as inflammatory cytokines [43,44]. Therefore, the interaction of NEMO and RIP1 is indispensable for the activation of the NF-κB signaling pathway. Blocking the binding of NEMO to polyubiquited RIP1 could inhibit the activation of IKK [62,63,64]. In this study, we found the interaction between polyubiquitinated RIP1 and NEMO induced by *S. aureus* infection was inhibited by acetylharpagide pretreatment in Raw264.7 cells. Thus, acetylharpagide inhibits the activity of NF-κB upon *S. aureus* infection by blocking polyubiquitinated RIP1 and NEMO interaction, but the mechanism needs further study.

## 4. Materials and Methods

### 4.1. Chemicals and Reagents

Six to eight week-old male C57BL6 mice (weight: 18–22 g) were obtained from the model animal research center of Nanjing University (Nanjing, China). Raw264.7 cell line (TIB-71) and USA300 (BAA-1717) were purchased from American Type Culture Collection (Manassas, VA, USA). RPMI1640 medium and lipofectamine 3000 were purchased from Thermo Scientific (Waltham, MA, USA). Penicillin-streptomycin solution, Ethylene Diamine Tetraacetic Acid (EDTA)-trypsin, dimethyl sulfoxide side (DMSO), wright stain, hematoxylin-eosin stain kit and nuclear protein extraction kit were purchased from Solarbio (Beijing, China). Acetylharpagide (CAS No. 6926-14-3, Item No. S9474) was purchased from Selleck (Houston, TX, USA). Polyvinylidene difluoride (PVDF) membrane and protein A/G agarose slurry were purchased from Millipore (Billerica, MA, USA). Evans blue dye was purchased from Sigma-Aldrich (St. Louis, MO, USA). Enzyme-linked immunosorbent assays (ELISA) kits were purchased from R&D (Minneapolis, MN, USA). P65, IκBα, phospho IκBα, NEMO, RIP1, α-tubulin and Histone H3 antibodies were purchased from Proteintech Group (Chicago, IL, USA). Dual-luciferase reporter assay system was purchased from Promega (Madison, WI, USA). TransStart Green qPCR SuperMix was purchased from TransGene Biotechnology (Beijing, China). Cell lysis buffer was purchased from Beyotime Biotechnology (Shanghai, China). Trizol reagent was purchased from Invitrogen (Carlsbad, CA, USA).

### 4.2. Animals

C57BL6 mice were preserved in a sterilized standard cage at 25 °C under a regular 12/12 h light and dark cycle, and supplied food and water randomly.

USA300 were cultivated in tryptic soy broth (TSB) medium overnight at 37 °C, and then re-inoculated into new TSB medium at 10:1. When it reached the logarithmic phase, pelleted and resuspended in phosphate buffered saline (PBS) to 1 × 10^9^ colony forming units (CFU)/mL. For the lung infection, mice were injected with 4% chloral hydrate intraperitoneally at a dose of 0.1 mL/10g for anesthesia. 2 × 10^8^ CFU of *S. aureus* or same volume of PBS was slowly dropped into the trachea through a self-made blunt-head syringe. Then kept the mouse upright for 1 min to distribute the bacterial solution evenly on both lungs. The experimental mice were grouped randomly as follows: (1) group PBS: mice were injected intraperitoneally with DMSO 2 h before PBS infection; (2) group SA: mice were injected intraperitoneally with DMSO 2 h before *S. aureus* infection; (3) group 5 mg/kg acetylharpagide + SA: mice were injected intraperitoneally with 5 mg/kg of acetylharpagide 2 h before *S. aureus* infection; (4) group 10 mg/kg acetylharpagide + SA: mice were injected intraperitoneally with 10 mg/kg of acetylharpagide 2 h before *S. aureus* infection; (5) group 20 mg/kg acetylharpagide + SA: mice were injected intraperitoneally with 20 mg/kg of acetylharpagide 2 h before *S. aureus* infection; (6) group 40 mg/kg acetylharpagide + SA: mice were injected intraperitoneally with 40 mg/kg of acetylharpagide 2 h before *S. aureus* infection. Acetylharpagide was dissolved in DMSO as a stock solution at a concentration of 200 mM. For in vivo/animal studies, the stock solution was suspended in sterile saline at a total volume of 200 μL per mouse. Taking the maximum concentration of 40 mg/kg injected into mice (18–22 g) as an example, it took about 10 μL of the stock solution to be diluted to 200 μL, so the final DMSO concentration did not exceed 5%. Control mice were injected with 5% DMSO. During the whole experiment, no mice showed obvious abnormal performance. For in vitro assays, the stock solution was suspended in cell culture medium in 2 mL for each well of a 6-well plate. Taking the maximum concentration of 20 μM as an example, it took 0.2 μL of stock solution to be diluted to 2 mL for each well, so the final DMSO concentration did not exceed 0.01%. Control sample was treated with 0.2 μL DMSO.

The survival of infected mice was monitored daily. The whole lung was harvested 12 h after *S. aureus* infection and homogenized for evaluating bacterial loads and MPO activity. BAL fluid was harvested for examining the concentration of cytokines and total protein, and the number of neutrophils. For lung histomorphology analysis, the lungs were fixed with 4% paraformaldehyde, dehydrated gradually with gradient ethanol, and then embedded in paraffin. The wax block was sliced with a thickness of 5 μm, and finally hematoxylin-eosin staining was performed.

The methods were conducted according to the approved guidelines and every effort was made to minimize suffering. All experimental protocols were approved by the Animal Experiment Committee of Nanjing University (SYXK (SU) 2019-0056, 2019.12.16).

### 4.3. Western Blot

Cell culture medium was removed and the cells were washed three times with PBS. 100 μL cell lysis buffer was added to each well of a 6-well plate. Cells were lysed on ice for 10 min and moved into a 1.5 mL centrifuge tube and centrifuged at 12,000 rpm for 10 min, the supernatant were collected and separated by sodium dodecylsulphate polyacrylamide gel electrophoresis. After that, the proteins on the gel were transferred to a PVDF membrane, which was then kept in 5% skimmed milk for 60 min. Next, the blocked membrane was coated with primary antibodies for p65, IκBα and phospho IκBα. It was then washed with phosphate buffered saline tween (PBST) buffer on a shaker for 10 min, and repeated twice. The washed membrane was further coated with a horseradish peroxidase-labeled antibody for 1 h. It was then washed with PBST buffer for 10 min, and repeated twice. Finally, the signal was detected by Tanon chemiluminescence imaging system.

### 4.4. Pulmonary Vascular Permeability and Edema

Evans blue dye can bind to albumin in plasma. When pulmonary capillaries leak, albumin that is bound to evans blue dye will penetrate into the lung tissue. The amount of evans blue dye in the lung can be measured by chemical colorimetry to reflect the permeability of blood vessels. In brief, mice were injected with 30 mg/kg of evans blue dye through the tail vein 1 h before completing the experiment. Once the mice were sacrificed at the indicated times, cardiac perfusion was performed until the outflow was completely clean. The lungs were then removed, immersed in dimethylformamide and kept at 60 °C for 24 h, and then centrifuged at 12,000 rpm for 30 min. The optical density value of the supernatant was detected at a wavelength of 620 nm with a spectrophotometer. The evans blue dye concentration is calculated based on its standard curve in dimethylformamide.

The wet/dry weight ratio is assessed to show the lung tissue water content. The lungs were collected and weighed, and this weight was recorded as the wet weight. Then the lungs were placed in 60 °C for 48 h and weighed again, and this weight was recorded as dry weight.

### 4.5. Cytokines and Neutrophils in BAL Fluid

To collect BAL fluid, mice were injected with 4% chloral hydrate intraperitoneally at a dose of 0.1 mL/10 g for anesthesia. Inserted a self-made blunt-head syringe into the trachea of the mouse and ligated the trachea to prevent the backflow. Gently injected 0.3 mL of sterile saline, then slowly withdrew. Three times in total. The collected liquid is BAL fluid. BAL fluid was treated with red blood cell lysis buffer and centrifuged at 1000 rpm for 5 min, the supernatant were applied for ELISA to detect the concentration of MIP-2, IL-1β, TNF-α, IL-6, MCP-1 and TGF-β. The pellet was resuspended in PBS and the total cell number was counted. The number of neutrophils were counted with wright stain.

### 4.6. Bacterial Counts

The lungs were removed aseptically, weighed and ground with a sterilized tissue grinder in 1 mL of sterilized PBS. The homogenate was diluted by 10-fold gradient and serially diluted to 10^8^. 100 µL of the tissue suspension was smeared on tryptic soy agar medium and kept at 37 °C for 24 h. Bacteria were counted and calculated as CFU per g of lung.

### 4.7. Coimmunoprecipitation Assay

Cell culture medium was removed and the cells were washed three times with PBS. 500 μL cell lysis buffer was added to each 60 mm dish. Cells were lysed on ice for 10 min and moved into a 1.5 mL centrifuge tube and centrifuged at 12,000 rpm for 10 min, the supernatant were collected. For immunoprecipitation, 500 μL of the supernatant was matched with 0.5 μg of antibody plus 30 μL of a 50% slurry of protein A/G agarose at 4 °C overnight. The precipitation was washed three times with PBS, resuspended in 20 μL SDS loading buffer and underwent immunoblotting.

### 4.8. Dual Luciferase Reporter Assay

Raw264.7 cells were transfected with a NF-κB luciferase reporter plasmid and infected with *S. aureus* as described above. Each sample was co-transfected with 20 ng pRL-TK Renilla luciferase reporter plasmid as a control reporter gene. Dual-luciferase reporter assay system was used to detect the luminescence, and NF-κB activity is presented by the ratio of firefly luminescence to renilla luminescence.

### 4.9. Quantitative Real-Time PCR

This experiment was carried out as previously described [65]. 1 mL Trizol reagent was added to each well of a 6-well plate. Cells were lysed at room temperature for 5 min, and then moved into a 1.5 mL centrifuge tube. Then added 200 μL chloroform, shook vigorously for 15 s, placed the tube on ice for 3 min, and centrifuged at 12,000 rpm for 10 min. Then aspirated the upper liquid into a new tube, added an equal volume of isopropanol, mixed well, placed the tube on ice for 10 min, and centrifuged at 12,000 rpm for 10 min. Discarded the supernatant, added 1 mL 75% alcohol to the tube, and then centrifuged at 12,000 rpm for 5 min. The precipitation was RNA.

Reverse transcriptase was then used to reverse-transcribe the RNA into cDNA, which was then subjected to quantitative PCR with the CFX Connect Real-Time PCR Detection System under the following conditions: 94 °C for 30 s, then 40 cycles at 94 °C for 5 s and 60 °C for 30 s. The mRNA levels of specific genes were normalized to β-Actin. The specific primer sequences for IL-1β, MCP-1, TNFα, MIP-2, IL-6 and TGF-β are listed in Supplementary Table S1.

### 4.10. Extraction of Cytoplasm and Nuclear Proteins

Nuclear protein extraction kit was used to separate nuclear and cytoplasmic proteins. Cells were first lysed by plasma protein extraction reagent. Added 100 μL plasma protein extraction reagent to each sample. Cells were lysed on ice for 10 min and centrifuged at 12,000 rpm for 10 min, the supernatant was the cytoplasmic protein and the precipitation was the nucleus. Aspirated the supernatant as completely as possible, then the nucleus was lysed by the nuclear protein extraction reagent. Added 50 μL nuclear protein extraction reagent to each sample. The nucleus was lysed on ice for 10 min and centrifuged at 12,000 rpm for 10 min, the supernatant was the nuclear protein.

### 4.11. Statistical Analysis

Data are expressed as the mean ± SD, and Student’s t-test was used to determine the statistically significant differences between mean values. Survival was analyzed using the Kaplan-Meier method and statistical analyses were performed using the log-rank test. * *p* < 0.05, ** *p* < 0.01, *** *p* < 0.001, and not significant, NS > 0.05.

## Supplementary Materials

The following are available online, Table S1: Primer sequences.
