# Supplementary material for: Acetylharpagide Protects Mice from Staphylococcus Aureus-Induced Acute Lung Injury by Inhibiting NF-κB Signaling Pathway
Source: Molecules. 2020 Nov 25;25(23):5523. doi: 10.3390/molecules25235523 (PMC7728067; doi:10.3390/molecules25235523)
Supplement: Supplementary file 1 [file molecules-25-05523-s001.pdf]

Supplementary Materials

Acetylharpagide protects mice from *Staphylococcus aureus*-induced acute lung injury by inhibiting NF-κB signaling pathway

ZhaoXin Zhang [1], Yun Wang [1], YaTing Shan [1] and Wu Yin [1,*]

[1] The State Key Lab of Pharmaceutical Biotechnology, College of life Sciences, Nanjing University, Nanjing 210023, China

* Correspondence: wyin@nju.edu.cn; Tel.: 13914761940

Table S1. Primer sequences

IL-1β-F	AATCTCGCAGCAGCACAT
IL-1β-R	ATCTCGGAGCCTGTAGTG
MCP-1-F	AGCAAGATGATCCCAATG
MCP-1-R	TGAAGACCTTAGGGCAGA
TNFα-F	TCTCATTCCTGCTTGTGGC
TNFα-R	GGCAGCCTTGTCCCTTGA
MIP-2-F	ACCAACCACCAGGCTACA
MIP-2-R	CTTCAGGGTCAAGGCAAA
IL-6-F	GTTGCCTTCTTGGGACTG
IL-6-R	TTTCCACGATTTCCCAGA
TGF-β-F	AGCGGACTACTATGCTAAAGAG
TGF-β-R	GCCACTCAGGCGTATCAG
β-Actin-F	GTTACCAACTGGGACGACA
β-Actin-R	CGACCAGAGGCATACAGG
